# Identification of key biomarkers in ischemic stroke: single-cell sequencing and weighted co-expression network analysis

**DOI:** 10.18632/aging.204855

**Published:** 2023-07-06

**Authors:** Jiali Tao, Xiaochen Xie, Man Luo, Qingsong Sun

**Affiliations:** 1 Department of Emergency Medicine, The Affiliated Huaian No. 1 People’s Hospital of Nanjing Medical University, Huaian 223300, People’s Republic of China; 2 Department of Respiratory Medicine, The Affiliated Huaian No. 1 People’s Hospital of Nanjing Medical University, Huaian 223300, People’s Republic of China

**Keywords:** ischemic stroke, biomarkers, transcriptome, analysis, bioinformatics

## Abstract

Purpose: At present, there is a lack of accurate early diagnostic markers for ischemic stroke.

Methods: By using dimensionality reduction cluster analysis, differential expression analysis, weighted co-expression network analysis, protein-protein interaction network analysis, cell heterogeneity and key pathogenic genes were identified in ischemic stroke. Immunomicroenvironment analysis was used to explore the immune landscape and immune associations of key genes in ischemic stroke. The analysis platform we use is R software (version 4.0.5). PCR experiments were used to verify the expression of key genes.

Results: Single cell sequencing data in ischemic stroke can be annotated as fibroblast cells, pre-B cell CD34, neutrophils cells, bone marrow (BM), keratinocytes, macrophage, neurons and mesenchymal stem cells (MSC). By the intersection of differential expression analysis and WGCNA analysis, 385 genes were obtained. Gene ontology (GO) and Kyoto Encyclopedia of Genes and Genomes (KEGG) enrichment analysis showed that these genes were highly correlated with multiple functions and pathways. Protein-protein interaction network analysis revealed that MRPS11 and MRPS12 were key genes, both of which were down-regulated in ischemic stroke. The Pseudo-time series analysis found that the expression of MRPS12 decreased gradually with the differentiation of pre-B cell CD34 cells in ischemic stroke, suggesting that the downregulation of MRPS12 expression may play an important role in ischemic stroke. At last, PCR showed that MRPS11 and MRPS12 were significantly down-regulated in peripheral blood of patients with ischemic stroke.

Conclusions: Our study provides a reference for the study of pathogenesis and key targets of ischemic stroke.

## INTRODUCTION

Ischemic stroke is a medical condition that occurs when the blood flow to a part of the brain is blocked, typically by a blood clot [1]. This lack of blood flow can cause brain cells to die, leading to a range of symptoms, such as numbness or weakness on one side of the body, difficulty speaking or understanding speech, and severe headache [2]. Numerous studies have identified various risk factors for ischemic stroke, including high blood pressure, smoking, diabetes, and high cholesterol [3]. Researchers continue to investigate how these and other risk factors contribute to the development of stroke and how they can be addressed to prevent stroke [4]. Advances in imaging technology have allowed researchers to better understand the mechanisms behind ischemic stroke [5]. For example, magnetic resonance imaging (MRI) can show the extent of damage to the brain following a stroke and help doctors determine the best course of treatment [6]. There are several treatments available for ischemic stroke, including thrombolytic therapy (using medication to dissolve the blood clot) and mechanical thrombectomy (using a device to physically remove the clot) [7]. Researchers are continuing to refine these treatments to improve their effectiveness and reduce the risk of complications. Preventing stroke is a major focus of research in this field, and several strategies have been developed to reduce the risk of ischemic stroke [8]. These include lifestyle changes such as exercise and healthy eating, as well as medications such as blood thinners and cholesterol-lowering drugs [8].

Overall, research into ischemic stroke is ongoing and has made significant progress in recent years. While there is still much to study the pathogenesis and early robust markers of ischemic stroke for improved outcomes for those affected by stroke [9]. Transcriptome analysis using sequencing technology is a powerful tool in the study of ischemic stroke [9]. It allows researchers to investigate changes in gene expression following a stroke and to identify the specific genes and signaling pathways involved in the development of stroke and the recovery process [10]. Transcriptome analysis can be used to identify potential biomarkers of ischemic stroke, reveal new therapeutic targets for the treatment of stroke, and provide insight into the underlying mechanisms of stroke pathophysiology [11]. Additionally, transcriptome analysis can help tailor treatment to individual patients by identifying subgroups with different molecular profiles [11]. Recent studies have yielded promising results, and further research is likely to uncover even more insights into this complex condition.

The development of high-throughput sequencing technology, also known as next-generation sequencing, has revolutionized the field of genomics by allowing rapid and cost-effective sequencing of entire genomes and transcriptomes [12]. Transcriptome sequencing, also known as RNA sequencing (RNA-seq), is a powerful tool for analyzing gene expression and has become increasingly popular in the study of ischemic stroke [13]. RNA-seq allows researchers to identify changes in gene expression in response to various stimuli, including stroke, and provides a comprehensive view of the transcriptome [14–16].

In this study, we analyzed single-cell sequencing data and high-throughput sequencing data for ischemic stroke, combined with a variety of methods, including dimensionality reduction clustering and weighted co-expression network analysis (WCGNA), and we found that MRPS11 and MRPS12 are robust markers of ischemic stroke, providing reference for its prevention and treatment.

## METHODS

### Single cell sequencing data download and quality control

For a vast variety of disorders, the GEO database contains single-cell sequencing and transcriptome sequencing data. The blood single cell sequencing dataset GSE174574 on ischemic stroke, which included three mouse model of middle cerebral artery occlusion (MACO) with ischemic stroke and three normal control samples with sham operation, was downloaded for this investigation [9]. The data was then processed and analyzed using the “Seurat” R package. These were the criteria for including genes in further analysis: 1. Genes that are expressed in three or more cells. 2. Genes that have an expression level greater than 200. The following are the criteria for include cells: 1. Gene expression fluctuates between 200 and 4000; 2. Less than 10 percent of the genes are mitochondrial genes.

### Processing of single cell sequencing data

We first standardize the data using the “LogNormalize” method of the “Seurat” package. The number of high-variable genes was then set to 3000, and the setting technique was changed to “vst” to conduct additional research on high-variable genes. The “Seurat” package’s SCTransform function was used to recombine data from several samples and remove cell cycle and mitochondrial interference. The data dimensions were reduced by using the UMAP technique with dims set to 30. The cells were clustered using the “KNN” algorithm, with the following settings: k.Paam = 30, dims = 20, resolution = 1.0, random.seed = 2023. Then, the annotations on cells were made using “SingleR” and the CellMarker website (http://117.50.127.228/CellMarker/index.html).

### Transcriptome data download and processing

Ischemic stroke-related blood transcriptome data sets GSE16561 and GSE58294 were downloaded from the GEO database, and expression matrices were standardized for subsequent analysis [17, 18]. A total of 63 samples were included in GSE16561, including 24 normal control samples and 39 stroke samples. A total of 92 samples were included in GSE58294, including 23 normal samples and 69 stroke samples. All data are standardized by log2 to facilitate subsequent analysis.

### Weighted gene coexpression network analysis (WGCNA)

Gene modules connected to ischemic stroke were sought after using WGCNA analysis. The soft threshold range were set as follows: step size is 1 for consecutive integers from 1 to 10; step size is 2 for consecutive integers from 12 to 20. To choose a good soft threshold, we utilized the “WGNCA” package’s pickSoftThresgold function. Set deepSplit to 2, the minimum number of module genes to 200, then grouped the genes into various modules. The following modules are combined with the truncation value of 0.3 at the same time. And lastly, each module was linked to a phenotype.

### Gene ontology (GO) enrichment analysis

GO (Gene Ontology) is a database produced by the Gene Ontology Consortium, seeking to establish a database relevant to various species, which can define and describe the activities of genes and proteins and is applicable to various species. Biological Process (BP), Cellular Component (CC), and Molecular Function (MF) are the three categories. The GO enrichment analysis was performed using the enrichGO function from the ClusterProfile package. We extract the top 10 signaling pathways with the smallest *p-*value for display.

### Kyoto Encyclopedia of Genes and Genomes (KEGG) enrichment analysis

The KEGG (Kyoto Encyclopedia of Genes and Genomes) database included metabolic pathways database, hierarchical categorization database, gene database, genome database, etc. It was a database that systematically analyzed gene functions, genomic information, and functional information. By comparing the examined genes with the pathway gene set in the database, the enriched pathways were produced. The KEGG enrichment analysis employed the ClusterProfile R package's enrichKEGG function. We extract the top 10 signaling pathways with the smallest *p* value for display. *p* < 0.05 and the Log |FC| > 0 are thought to be statistically significant.

### Protein-protein interaction (PPI) network analysis

Proteins that interact with one another and take part in numerous parts of life processes, such as biological signal transduction, gene expression regulation, energy and substance metabolism, and cell cycle regulation, make up protein-protein interaction networks (PPI). Understanding the working principle of proteins in biological systems, the reaction mechanism of biological signals, the metabolism of energy and materials in particular physiological states like diseases, as well as the functional relationships between proteins, all depend on systematic analysis of the interaction between a large number of proteins in biological systems. In this study, the STRING database (https://cn.string-db.org/) was used for PPI analysis. The top 20 genes’ interactions were shown using the cytoHubba plugin of the Cytoscape program, which was used to visualize the results.

### Pseudo-time series analysis

To examine the state of cell differentiation, the “monocle2” software was frequently used for cell differentiation trajectory analysis. In this study, we screened genes with average expression more than 0.1 for further investigation and chose a particular cell subpopulation for this analysis. We used the “reduceDimension” function to sort the cell differentiation trajectory and decrease the dimension with the parameters max components = 2, method = DDRTree, and “reduceDimension” function. Cell differentiation was displayed using a UMAP map.

### PCR experiment

Peripheral blood samples from 30 patients with ischemic stroke and 30 healthy controls were separated by density gradient centrifugation. We then cleaved and homogenized the organs in Trizol (Vazyme Biotech, Nanjing, China), extracted RNA, and synthesized cDNA using reverse transcription reagent (Vazyme Biotech). PCR analysis was then performed using SYBR Green Premix reagent (Vazyme Biotech) and PCR system (Applied Biosystems, Foster City, CA, USA). Relative gene expression was calculated by 2^−ΔΔCt^ method. Supplementary Table 1 showed the sequence of primers we used.

### Statistical analysis

In single-cell sequencing data, we searched for differentially expressed genes associated with stroke by using the FindMarkers function of the “Seurat” package. In transcriptome data, WGCNA analysis was used to search for gene modules associated with stroke. The difference in gene expression between stroke and normal samples was analyzed using the rank sum test. All analyses were performed using R software, version 4.0.5. Unless otherwise noted, *p* < 0.05 was defined as statistically significant.

## RESULTS

Our entire workflow was summarized in Supplementary Figure 1.

### Single cell sequencing data analysis

**Figure 1:**
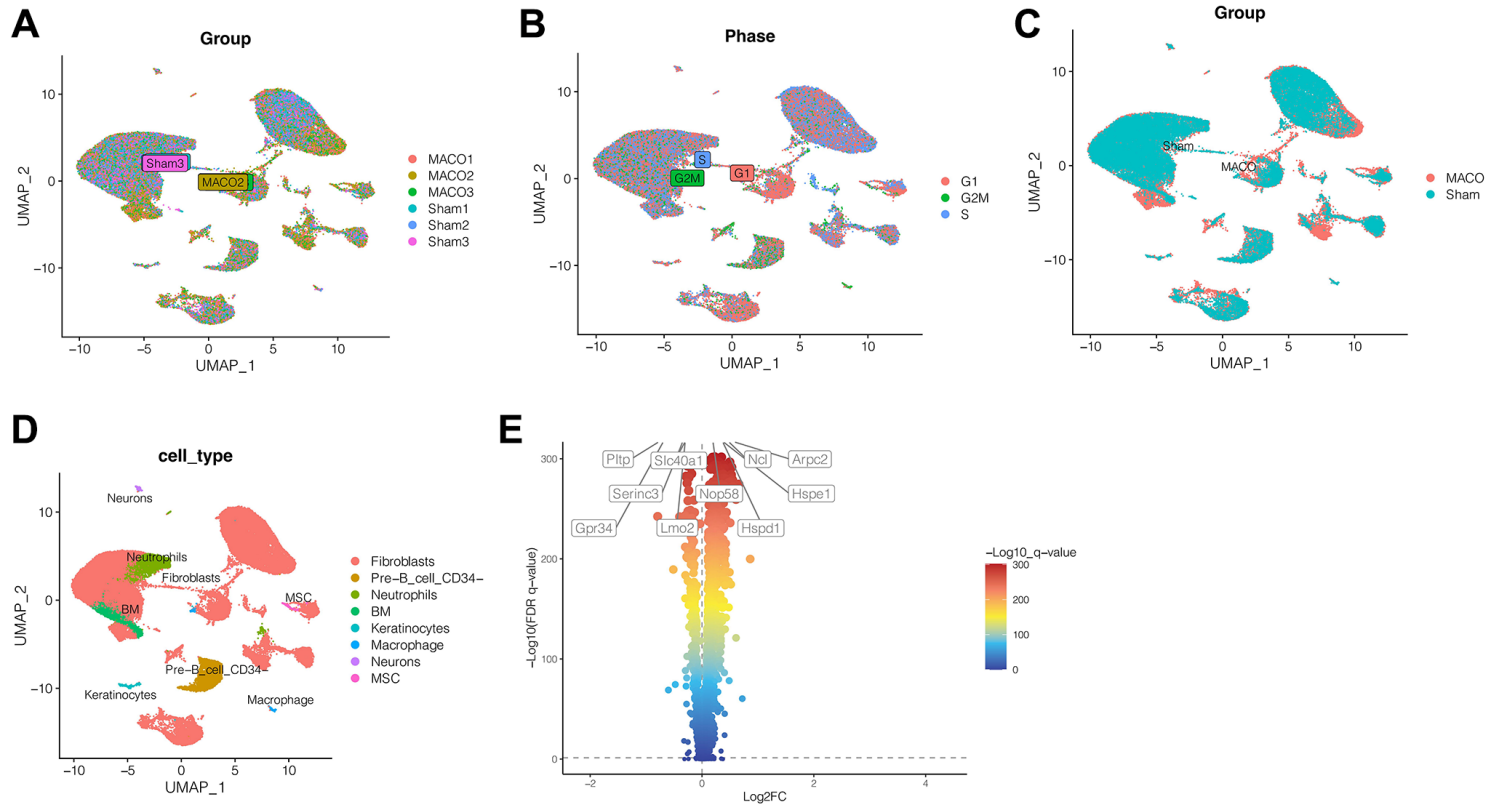
**Single cell sequencing analysis.** (**A**) No significant batch effect was observed. (**B**) The cell cycle had no effect on our subsequent analysis. (**C**) The heterogeneity between ischemic stroke group and normal control group. (**D**) 34 clusters were annotated as different cell types. (**E**) The first 5 genes that were significantly up-regulated were NCL, ARPC2, NOP58, HSPE1 and HSPD1, while the first 5 genes that were significantly down-regulated were PLTP, SLC40A1, SERINC3, LMO2 and GPR34. (Abbreviations: MACO: middle cerebral artery occlusion group; Sham: sham operation group).

After quality control of ischemic stroke and control samples, we obtained a total of 18,676 genes and 58,469 cells. As shown in Figure 1A, we found a fairly uniform distribution of cells in the three ischemic stroke samples and the three normal control samples, with no significant batch effect. As shown in Figure 1B, the cell cycle had no effect on our subsequent analysis. As shown in Figure 1C, the blue cells were cells in the normal control group, while the red cells were cells in ischemic stroke group, showing obvious heterogeneity among them. Through cluster analysis, we coclustered all the cells into 34 clusters. Then, we annotated the cells through SingleR and CellMarker website, as shown in Figure 1D. A total of 8 cell types have been annotated, namely fibroblast cells, pre-B cell CD34, neutrophils cells, BM, keratinocytes, macrophage, neurons and MSC. Through differential expressed gene analysis, ischemic stroke-related genes were found. As shown in Figure 1E, the first 5 genes that were significantly up-regulated were NCL, ARPC2, NOP58, HSPE1 and HSPD1, while the first 5 genes that were significantly down-regulated were PLTP, SLC40A1, SERINC3, LMO2 and GPR34.

### Weighted coexpression network analysis (WGCNA)

**Figure 2:**
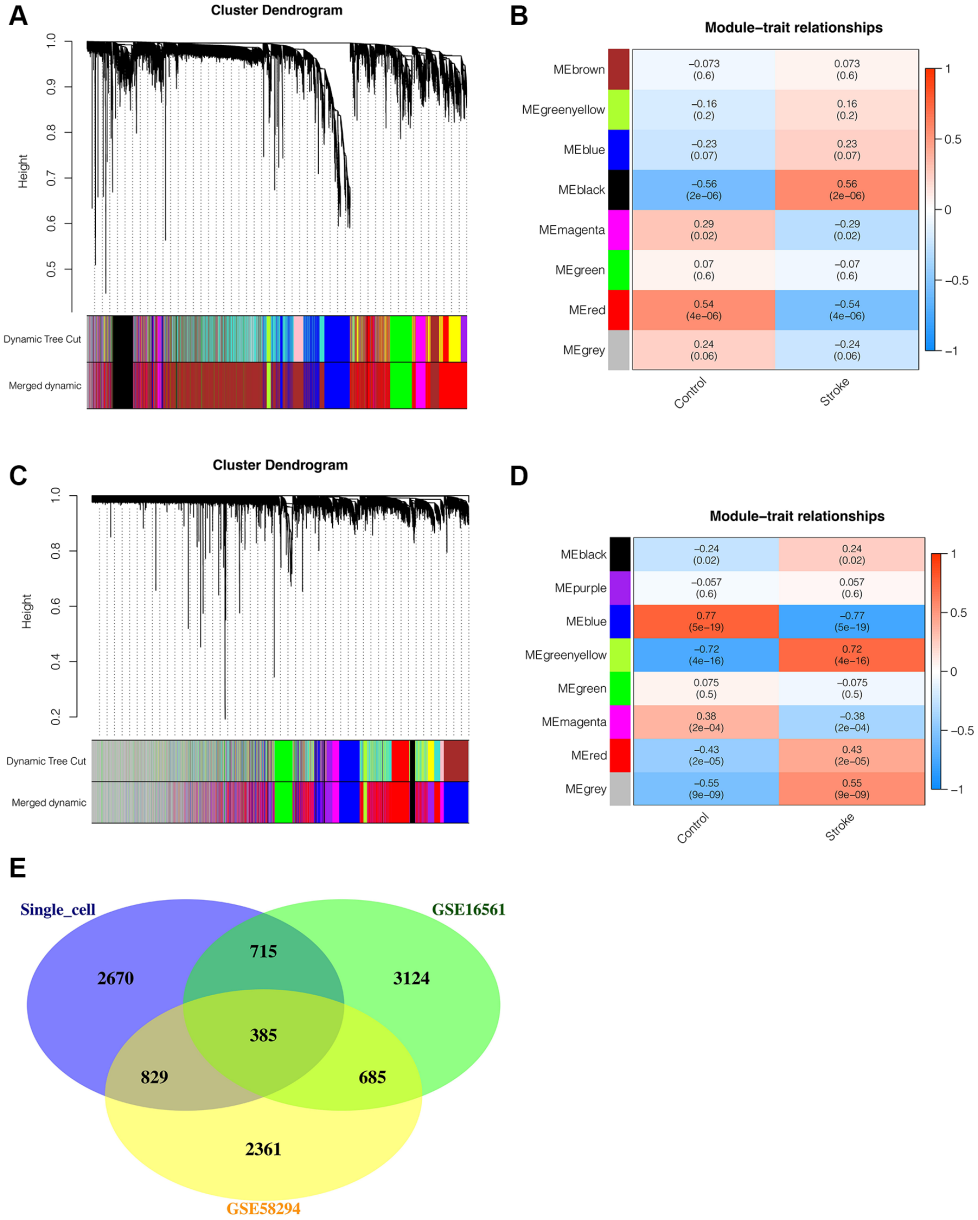
**Weighted coexpression network analysis (WGCNA).** (**A**, **B**) WGCNA in GSE16561. All genes were coclustered into 7 non-gray modules, among which black and red modules were most closely associated with ischemic stroke, and *p* < 0.05, with a correlation of 0.56 and 0.54, respectively. (**C**, **D**) WGCNA in GSE58294. All genes were coclustered into 7 non-gray modules, among which blue and greenyellow modules were most closely associated with ischemic stroke, and *p* < 0.05, with a correlation of 0.77 and 0.72, respectively. (**E**) The Venn intersection of genes obtained from single-cell analysis and genes obtained from transcriptome WGCNA analysis.

To further explore differences between ischemic stroke and normal control samples in transcriptome sequencing, WGCNA analysis was then performed. In the WGCNA of GSE16561, we found that the optimal soft threshold value was 7, and the data matched the power law distribution, which was suitable for subsequent analysis. As shown in Figure 2A, 2B, all genes were coclustered into 7 non-gray modules, among which black and red modules were most closely associated with ischemic stroke, and *p* < 0.05, with a correlation of 0.56 and 0.54, respectively. Therefore, the genes of the black and red modules were included in the follow-up analysis. There were 4909 genes in total, among which 1219 were in black module and 3690 were red genes. In the analysis of GSE58294 dataset, we found that the optimal soft threshold was also 7. As shown in Figure 2C, 2D, all genes were coclustered into 7 non-gray modules, among which blue and greenyellow modules were most closely associated with ischemic stroke, and *p* < 0.05, with a correlation of 0.77 and 0.72, respectively. Therefore, the genes of blue and greenyellow modules were included in the follow-up analysis, and there was a total of 4260 genes, including 3926 genes of black and 334 genes of red. In order to find genes relatively associated with ischemic stroke, the intersection of genes obtained from single-cell analysis and genes obtained from transcriptome WGCNA analysis was performed, as shown in Figure 2E, with 385 intersection genes in total.

### Enrichment analysis and protein-protein interaction network construction

**Figure 3:**
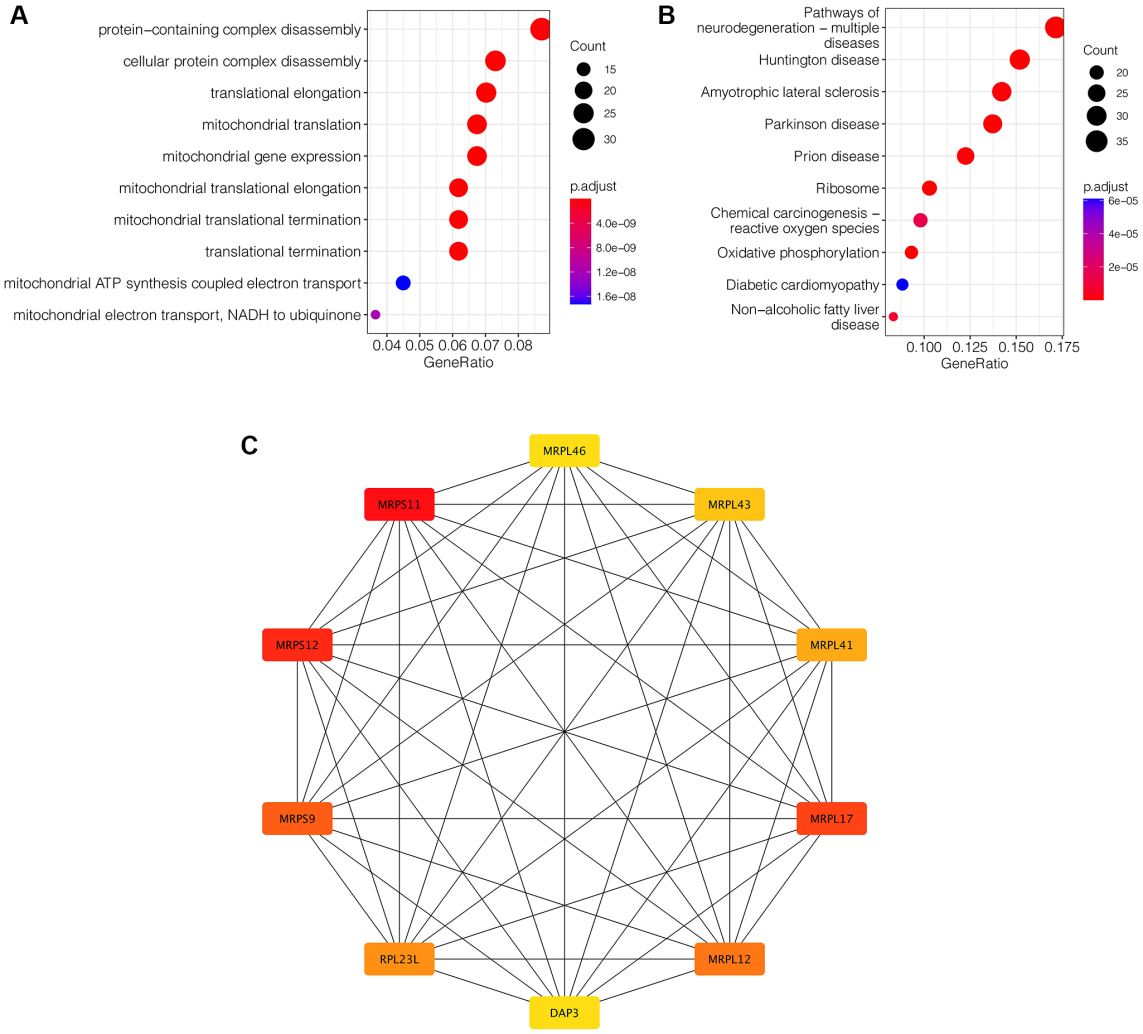
**Enrichment analysis and protein-protein interaction network construction.** (**A**) GO enrichment analysis. (**B**) KEGG enrichment analysis. (**C**) PPI network analysis. MRPS11 and MRPS12 are the two hub genes.

To further clarify the mechanism of action mediated by these 385 key genes in ischemic stroke, enrichment analysis was performed. As is shown in Figure 3A, GO enrichment analysis showed that these genes were mainly associated with protein-containing complex disassembly, cellular protein complex disassembly, translational elongation and other related functions. KEGG enrichment analysis showed that these genes were mainly neurodegeneration-multiple diseases, Huntington disease, Amyotrophic lateral sclerosis and other pathways (Figure 3B). Subsequently, in order to find the hub gene among these genes, PPI network analysis was conducted, as shown in Figure 3C. CytoHubba plugin allowed us to see the patterns of interactions between the top 20 genes and rank them from most important to least important. MRPS11 and MRPS12 were the two hub genes (Figure 3C).

### Expression analysis of hub genes

**Figure 4:**
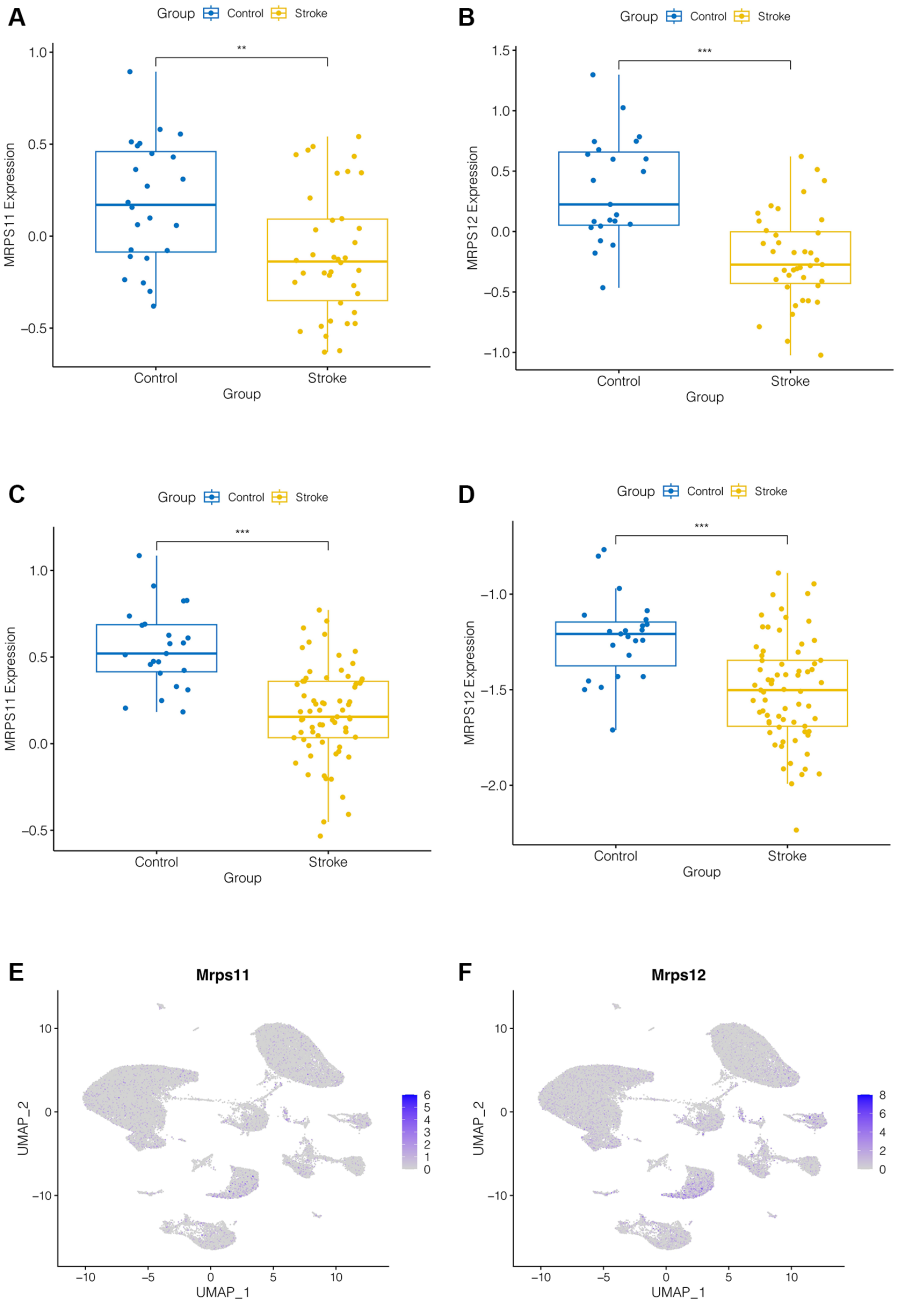
**Expression analysis of hub genes.** (**A**–**D**) MRPS11 and MRPS12 were down-regulated in ischemic stroke patients in the GSE16561 and GSE58294 data sets (*p* < 0.001). (**E**, **F**) Expression analysis of MRPS11 and MRPS12 in single cell data.

As shown in Figure 4A–4D, we found that both MRPS11 and MRPS12 were down-regulated in ischemic stroke patients in the GSE16561 and GSE58294 data sets (*p* < 0.001). And we found that, as shown in Figure 4E, 4F, expression analysis of MRPS11 and MRPS12 in single cell data showed that they were mainly in the pre-B cell CD34, especially MRPS12 was more obvious, suggesting that MRPS11 and MRPS12 may play an important role in ischemic stroke patients.

### Pseudo-time series analysis

**Figure 5:**
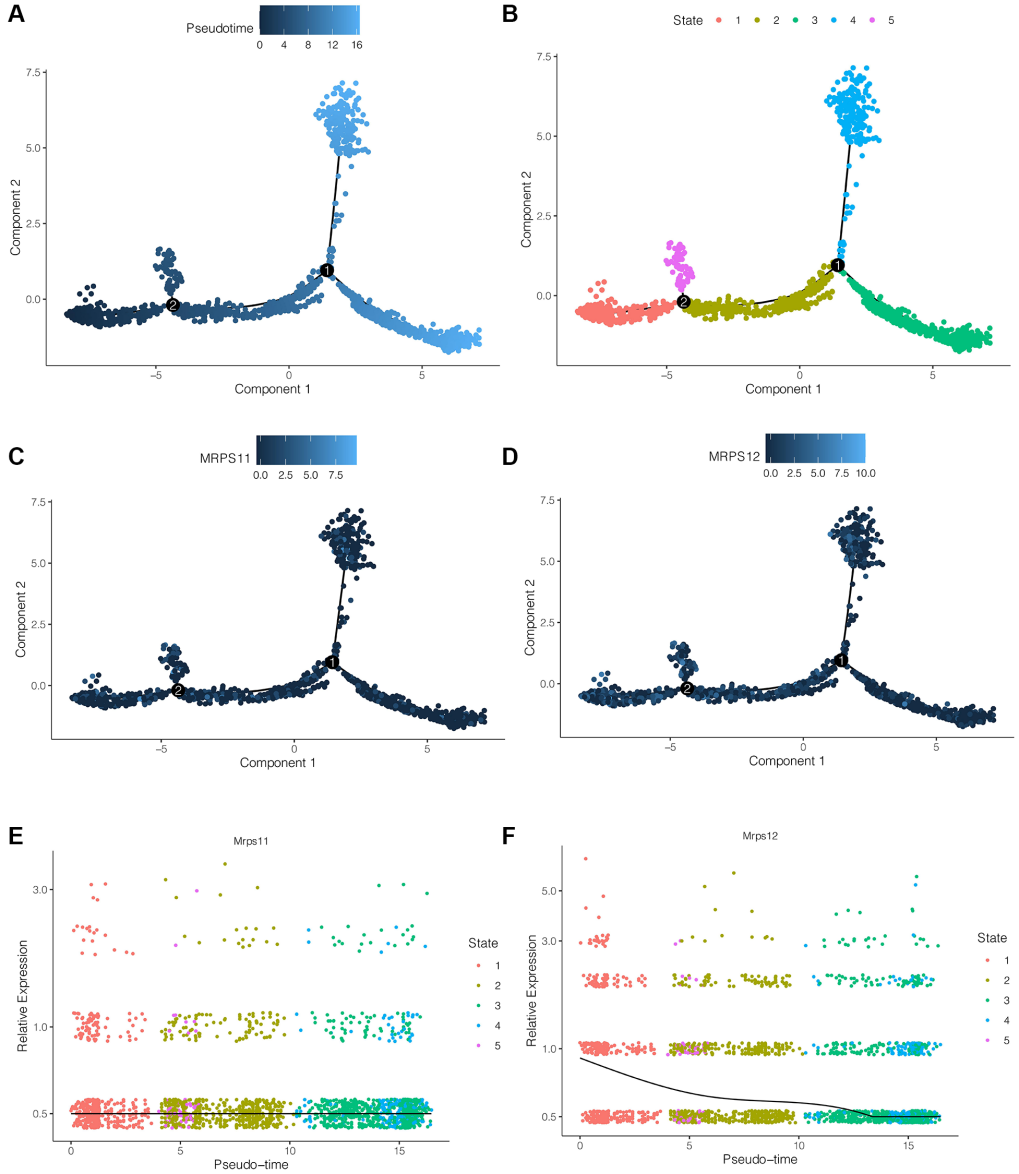
**Pseudo-time series analysis.** (**A**, **B**) Cells differentiation diagram. pre-B cell CD34 cells have five differentiation states, among which state 1 is early differentiation, and state 3 and state 4 is late differentiation. (**C**–**F**) During the differentiation of pre-B cell CD34 cells, the expression of MRPS11 did not change significantly, while the expression of MRPS12 showed a trend of gradual decline.

pre-B cell CD34 plays an important role in ischemic stroke, and the above analysis showed that both MRPS11 and MRPS12 were mainly expressed in pre-B cell CD34. Therefore, we extracted pre-B cell CD34 cells from ischemic stroke samples and used the “monocle2” package for pseudo-time series analysis. As shown in Figure 5A, 5B, cells differentiate from dark blue to light blue. pre-B cell CD34 cells have five differentiation states, among which state 1 is early differentiation, and state 3 and state 4 is late differentiation. As shown in Figure 5C–5F, during the differentiation of pre-B cell CD34 cells, the expression of MRPS11 did not change significantly, while the expression of MRPS12 showed a trend of gradual decline.

### Immune cell infiltration analysis

**Figure 6:**
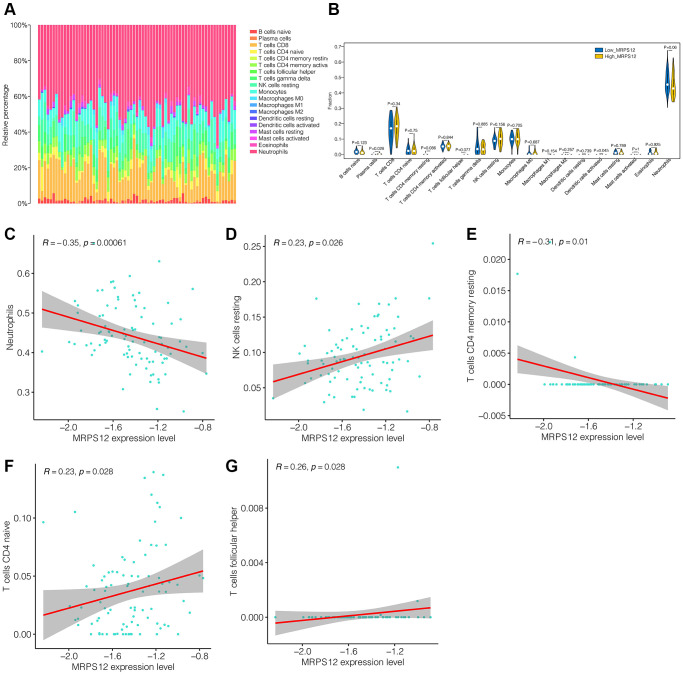
**Immune cell infiltration analysis.** (**A**) The immune infiltration landscape in ischemic stroke patients. (**B**) The infiltration levels of immune cells difference between the high-MRPS12 expression group and low-MRPS12 expression group. (**C**–**G**) Correlation analysis of the immune cells associated with MRPS12.

Through the above analysis, it is found that the down-regulation of MRPS12 may play an important role in the occurrence and development of ischemic stroke, and may be the target of its diagnosis and treatment. Subsequently, in transcriptome sequencing data, we further explored the association between MRPS12 and immune cells in GSE58294. Figure 6A showed immune infiltration in ischemic stroke patients. Neutrophils cells accounted for more of them. As shown in Figure 6B, the infiltration levels of plasma cells and dendritic cells activated were different between the high-MRPS12 expression group and low-MRPS12 expression group. Then in the correlation analysis, as shown in Figure 6C–6G, we found that the immune cells positively associated with MRPS12 included NK cells resting, T cells CD4 native and T cells follicular helper. Neutrophils and T cells CD4 memory resting were negatively correlated. This provided a reference for us to understand the mechanism of MRPS12.

### PCR to validate the expression of MRPS11 and MRPS12

**Figure 7:**
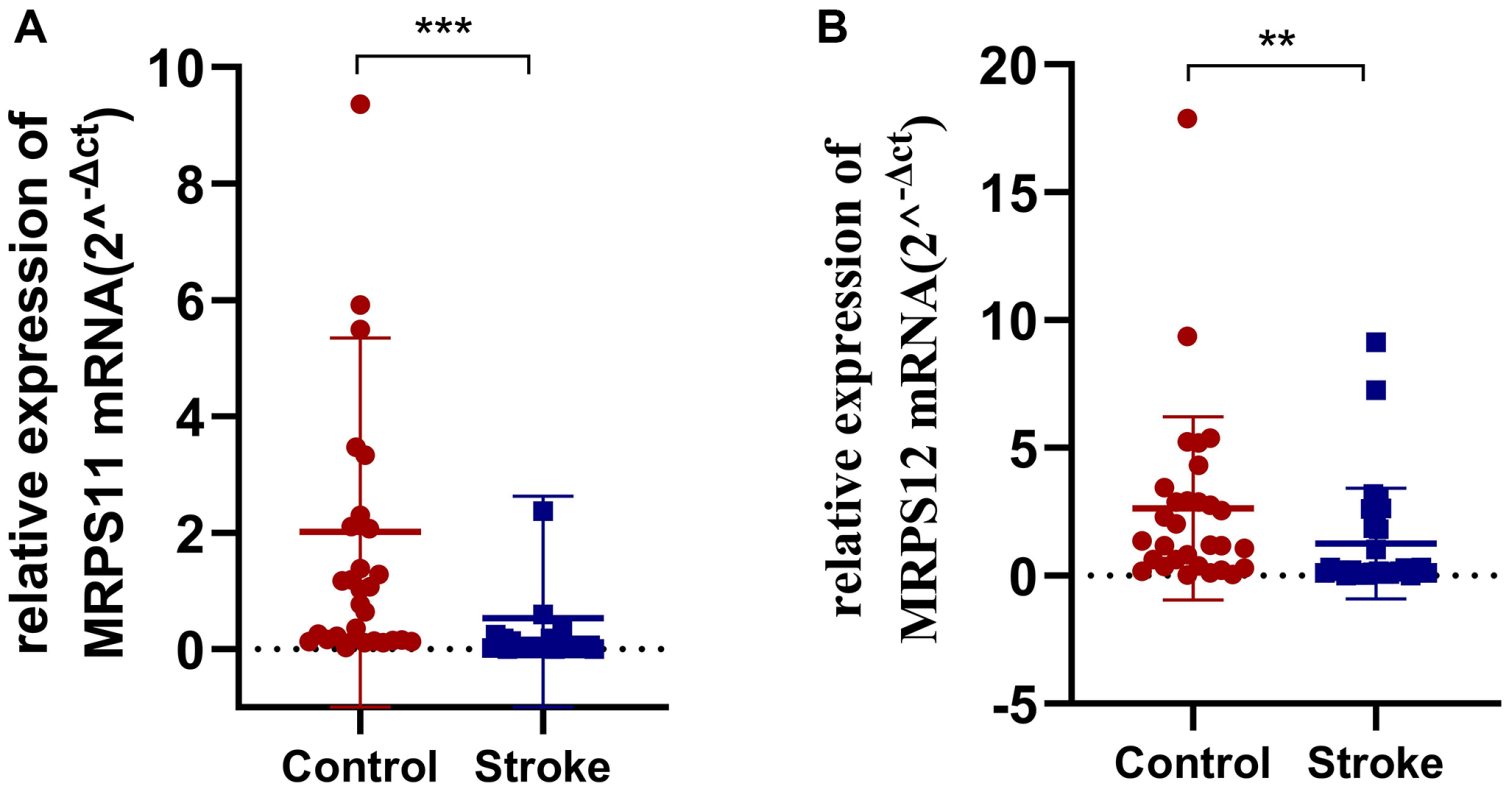
**PCR experiment.** (**A**, **B**) MRPS11 and MRPS12 were significantly down-regulated in peripheral blood of patients with ischemic stroke (^**^p < 0.01, ^***^p < 0.001).

Finally, in order to verify the expression of MRPS11 and MRPS12 in peripheral blood of patients with ischemic stroke, clinical samples were collected and PCR experiments were conducted. The results showed that compared with healthy controls, MRPS11 and MRPS12 were significantly down-regulated in peripheral blood of patients with ischemic stroke (Figure 7, ^**^P < 0.01, ^***^P < 0.001).

## DISCUSSION

Ischemic stroke is a medical emergency that requires rapid diagnosis and treatment [19]. Current diagnosis relies on clinical evaluation, imaging studies, and laboratory tests, with brain imaging being the gold standard [20]. The most effective treatments for ischemic stroke are thrombolytic therapy and mechanical thrombectomy, but they have a narrow time window for effectiveness and may not be appropriate for all patients [21]. Prevention through lifestyle changes and medication is crucial. However, limitations remain, such as the need for more effective preventative measures and new treatments for ischemic stroke, as well as the challenge of arriving at the hospital in time for effective treatment [22]. Further research is needed to develop new diagnostic and treatment approaches to improve outcomes for patients with ischemic stroke.

The present study identified MRPS11 and MRPS12 as robust markers in transcriptomic analysis of ischemic stroke. MRPS11 and MRPS12 have the potential to serve as biomarkers for ischemic stroke diagnosis, either alone or in combination with other biomarkers.

One potential application of MRPS11 and MRPS12 is as biomarkers for the diagnosis of ischemic stroke. By analyzing the expression of these genes, clinicians may be able to accurately diagnose ischemic stroke and distinguish it from other conditions with similar symptoms. Additionally, MRPS11 and MRPS12 could potentially serve as therapeutic targets for the treatment of ischemic stroke, as mitochondrial dysfunction has been implicated in stroke pathophysiology.

Further research is needed to validate the robustness of MRPS11 and MRPS12 as markers for ischemic stroke diagnosis and as therapeutic targets. However, the discovery of these genes as potential markers in transcriptomic analysis of ischemic stroke is a significant finding that has the potential to improve our understanding of this condition and lead to new diagnostic and treatment approaches.

MRPS11 and MRPS12 belong to the mitochondrial ribosomal proteins (MRPS) family, which is involved in the assembly of mitochondrial ribosomes and protein synthesis within the mitochondria [23–25]. Mitochondrial ribosomes are distinct from the ribosomes in the cytoplasm, and the MRPS family plays a critical role in the translation of mitochondrial DNA-encoded proteins [26].

Research on the MRPS family has focused on their role in mitochondrial function and their potential as therapeutic targets for various diseases [27]. Changes in the expression of MRPS genes have been linked to various conditions, including cancer, cardiovascular disease, and neurodegenerative diseases [28].

In particular, MRPS11 and MRPS12 have been implicated in the pathogenesis of cancer. A study found that the interaction between LncRNA ZFHX4-AS1 and MRPS11 may be related to the immune microenvironment in ovarian cancer and promote ovarian cancer progression [29]. Another study found that the expression of MRPS12 is positively correlated with the infiltration of macrophages and neutrophils in ovarian cancer, and MRPS12 is a potential oncogene and a promising prognostic candidate in ovarian cancer [30].

Multiomics analysis, a comprehensive approach that integrates multiple omics technologies such as genomics, transcriptomics, proteomics, and metabolomics, holds significant promise in the field of ischemic stroke research. By examining various molecular levels simultaneously, multiomics analysis enables a deeper understanding of the complex pathophysiological mechanisms underlying ischemic stroke. It allows the identification of key genetic variants, gene expression patterns, protein alterations, and metabolite profiles associated with stroke, providing valuable insights into the molecular pathways involved in the disease. This integrative approach can aid in the discovery of novel biomarkers for early diagnosis, prognosis, and personalized treatment strategies, ultimately leading to improved patient outcomes and the development of targeted therapeutic interventions for ischemic stroke.

Overall, the MRPS family, including MRPS11 and MRPS12, plays a critical role in mitochondrial function and has been linked to various diseases. The identification of MRPS11 and MRPS12 as potential markers in transcriptomic analysis of ischemic stroke highlights the importance of these genes in neurological disorders and their potential as therapeutic targets.

## Supplementary Materials

Supplementary Figure 1

Supplementary Table 1
