# Supplementary figures and images for: Identification of key biomarkers in ischemic stroke: single-cell sequencing and weighted co-expression network analysis
Source: Aging (Albany NY). 2023 Jul 6;15(13):6346–60. doi: 10.18632/aging.204855 (PMC10373980; doi:10.18632/aging.204855)

## SUPPLEMENTARY FIGURE

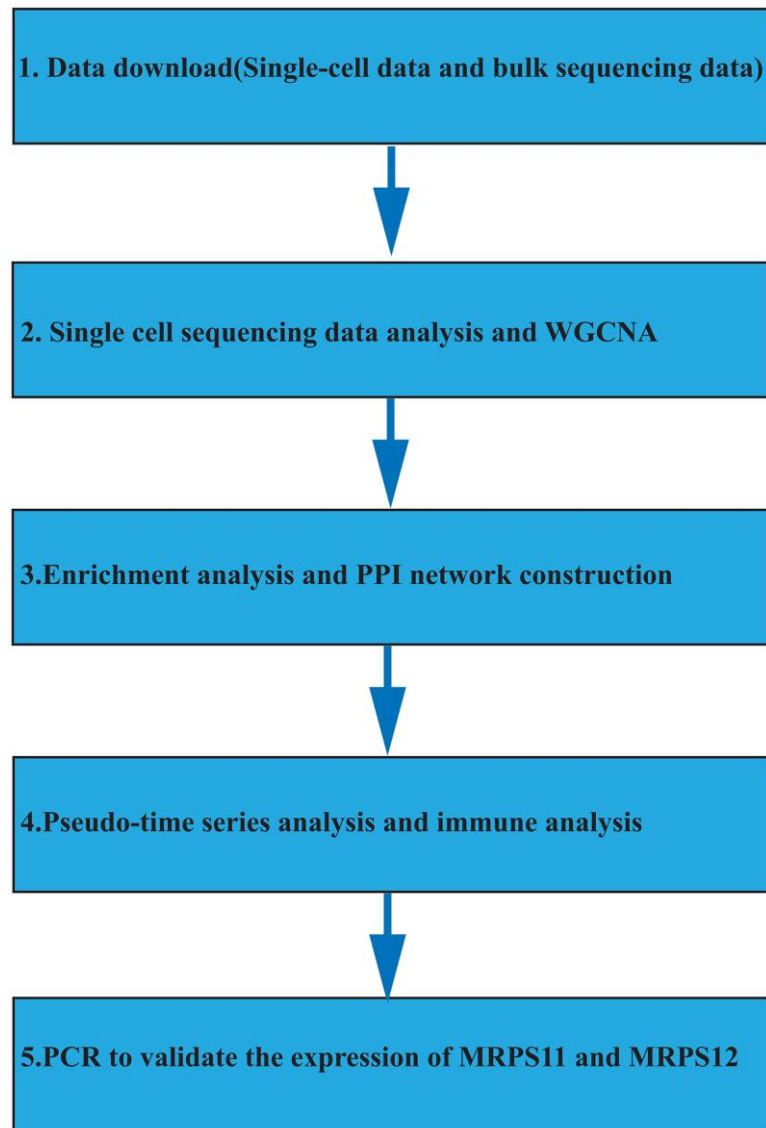

Supplementary Figure 1. The flow chart.

Supplement: Supplementary Figure 1 [file aging-15-204855-s001.pdf]
