# Supplementary material for: Identification of key biomarkers in ischemic stroke: single-cell sequencing and weighted co-expression network analysis
Source: Aging (Albany NY). 2023 Jul 6;15(13):6346–60. doi: 10.18632/aging.204855 (PMC10373980; doi:10.18632/aging.204855)
Supplement: Supplementary Table 1 [file aging-15-204855-s002.pdf]

SUPPLEMENTARY TABLE

Supplementary Table 1. Oligonucleotides used in research.

Oligonucleotides	Nucleotide sequence (5′–3′)
**Primer**	
GAPDH	Forward: GGCCTCCAAGGAGTAAGACC<br>Reverse: AGGGGAGATTCAGTGTGGTG
MRPS11	Forward: GGACTTGGCCCCAGACAG<br>Reverse: GCCGCGTTCTGTTCAACTTT
MRPS12	Forward: CCAGAAAGTCCTGAGAGCGG<br>Reverse: CAG0AGCTGGGCCACAAGTTA
